# Physical activity and mortality in patients with dementia: 2009–2015 National Health Insurance Sharing Service data

**DOI:** 10.1371/journal.pone.0301035

**Published:** 2024-05-15

**Authors:** Sanghun Nam, Ickpyo Hong, Min Seok Baek

**Affiliations:** 1 Department of Occupational Therapy, Graduate School, Yonsei University, Wonju, Republic of Korea; 2 Department of Occupational Therapy, College of Software and Digital Healthcare Convergence, Yonsei University, Wonju, Republic of Korea; 3 Department of Neurology, Wonju Severance Christian Hospital, Yonsei University Wonju College of Medicine, Wonju, Republic of Korea; Utah State University, UNITED STATES

## Abstract

The study aimed to investigate the survival rate of patients with dementia according to their level of physical activity and body mass index (BMI). A total of 5,789 patients with dementia were retrieved from the 2009–2015 National Health Insurance Sharing Service databases. Survival analysis was used to calculate the hazard ratio (HR) for physical activity and BMI. The study sample primarily comprised older adults (65–84 years old, 83.81%) and female (*n* = 3,865, 66.76%). Participants who engaged in physical activity had a lower mortality risk (HR = 0.91, *p* = 0.02). Compared to the underweight group, patients with dementia who had normal weight (HR = 0.86, *p* = 0.01), obesity (HR = 0.85, *p* = 0.03) and more than severe obesity (HR = 0.72, *p* = 0.02) demonstrated a lower mortality risk. This study emphasizes the significance of avoiding underweight and engaging in physical activity to reducing mortality risk in patients with dementia, highlighting the necessity for effective interventions.

## Introduction

Dementia is a debilitating condition affecting millions of people worldwide. It is characterized by a decline in cognitive function and can have a significant impact on individuals’ physical and mental well-being [1]. As the population ages, the prevalence of dementia is expected to rise, which highlights the need for effective interventions for patients with dementia [2].

Physical activity has been reported to be a potentially modifiable factor in determining the survival rates of patients with dementia [3]. Tolppanen, Solomon [4] found that higher levels of physical activity were associated with a reduced progression of dementia among patients with Alzheimer’s disease [4]. In addition, Scherder, Van Paasschen [5] found that physical activity was associated with better executive function in elderly individuals with mild cognitive impairment, which is often a precursor to dementia. Another previous study reported that higher levels of physical activity over the life course were associated with better cognitive performance in old age and may help to protect against cognitive impairment and dementia [6]. A systematic review also found that exercise programs may improve cognitive function and activities of daily living for individuals with dementia [7]. While the evidence is limited, it suggests that exercise may be a promising intervention for individuals with dementia. However, although many studies are reporting that physical activity reduces the progression of dementia and improves executive function in patients with dementia, there is a lack of research examining the risk of death between dementia patients and physical activity. In other words, there is a lack of research reporting on whether physical activity can reduce mortality in dementia patients.

In addition, body mass index (BMI) has also been reported as a potentially significant factor influencing the prevalence of patients with dementia [7]. Albanese, Launer [8] reported that higher BMI was negatively associated with prevalence in patients with dementia. However, the association between late-life dementia and BMI is multifaceted, and it could be influenced by various factors, including age, sex, and timing of BMI measurement. While both low and high BMI have been associated with an increased risk of dementia, high midlife BMI has been identified as a significant risk factor for dementia later in life [9–12]. However, previous studies have extensively investigated the role of BMI as a risk factor for dementia in the general population [13,14]. In addition, there has been limited focus on investigating the relationship between BMI and dementia, particularly among individuals diagnosed with dementia. Further studies are needed to investigate the complex association between BMI and dementia in older adults.

Given the potential impact of physical activity and BMI on the survival rates of patients with dementia, further research in this area is needed. Therefore, this study aimed to investigate the survival rate of patients with dementia according to physical activity and BMI. Understanding the complex interplay between these factors could lead to the development of more effective interventions to improve health outcomes for patients with dementia. The hypotheses for this study are as follows: Older adults with dementia who have high levels of physical activity are expected to exhibit a higher survival rate compared to those with low levels of physical activity. Additionally, older adults with dementia who have a normal BMI or higher BMI are anticipated to show a higher survival rate than those with an underweight BMI.

## Materials and methods

### Data sources

We utilized the 2009–2015 National Health Insurance Sharing Service (NHISS) databases provided by the National Health Insurance Corporation. NHISS, provided by the National Health Insurance Corporation in South Korea, offers a substantial advantage with its nationally representative large-scale dataset encompassing a wide array of health and medical information. Additionally, NHISS is widely utilized to support healthcare policies and conduct academic research, boasting high levels of data reliability and quality [15]. NHISS databases consist of health checkup cohort databases, sample cohort databases, senior cohort databases, customized research databases, health and disease indicators, and other data. In this study, we used the sample cohort database that was collected from one million Korean residents eligible for health insurance and medical assistance. The sample cohort included the Long-Term Care, Birth and Death, Health Examination, and Eligibility and Insurance databases. The NHISS data are collected under the Korean government oversight, encompassing mandatory insurance enrollment and medical records from healthcare institutions. Health authorities secure data for health statistics and research purposes through dedicated programs, utilizing healthcare information systems for the collection of medical records. The gathered information undergoes anonymization or processing by personal information protection policies. More detailed information about the NHISS databases is available at https://nhiss.nhis.or.kr.

**Fig 1:**
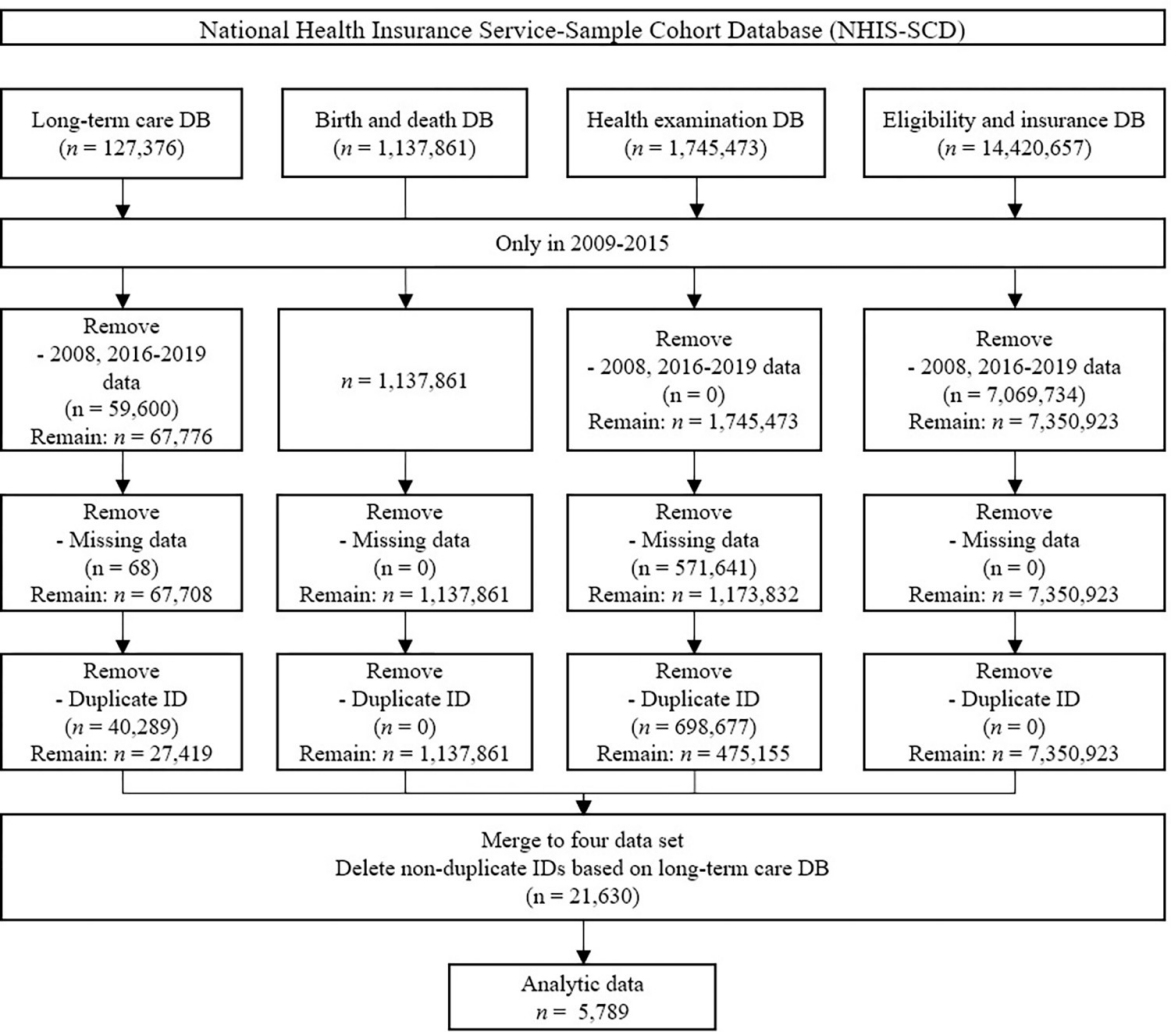
Cohort selection flow diagram. DB: Database, ID: Identification.

A sample cohort from the NHISS database was used to investigate the effect of physical activity status, physical activity frequency, and BMI on patients with dementia. In this study, written informed consent from the participants was not sought, as the data utilized herein consisted of pre-existing and anonymized information. This study was approved by the Yonsei University Institutional Review Board (#1041849-202304-SB-062-01) and met the research exemption criteria for the participating institutions. Furthermore, it is noteworthy that our research exclusively involved adult participants, thus obviating the requirement for parental or guardian consent. We accessed the shared data for analysis from February 2023 to March 2023. Furthermore, the shared data we utilized had individual participant-identifiable information removed, and we conducted our analysis using assigned numerical identifiers to ensure participant anonymity. The study variables were extracted from the sample cohort by removing missing values and duplicate individual identification numbers. Lastly, we extracted patients with dementia from the long-term care database. As a result, a total of 5,789 patients were included in the final analytic data (Fig 1).

### Independent variables

The independent variables were physical activity (high intensity, moderate intensity, or walking), number of physical activities during the past week, age, and BMI. Physical activity variables were extracted from the Health Examination database and categorized into three groups, such as high intensity, referring to vigorous physical activity lasting 20 minutes or more per week; moderate intensity, indicating moderate physical activity lasting 30 minutes or more per week; and walking physical activity, consisting of walking for 30 minutes or more per week. Physical activity was coded as 1 when one or more types of physical activity were performed in the past week and 0 when none were performed. The number of physical activities was the sum of each high-intensity, moderate-intensity, and walking physical activity (scores ranged from 0 to 2). Finally, the BMI value was extracted from the Health Examination database and coded into five groups using the Asia-Pacific BMI standard, including underweight (BMI < 18.4), normal weight (18.5 < = BMI < = 22.9), overweight (23 < BMI < 24.9), obesity (25 < BMI < 29.9), and severe obesity (BMI > 30) [16].

### Dependent variables

The death rates in the patients with dementia were extracted from the Birth and Death database. The variable of death date was used to code deceased patients with dementia as 1 and surviving patients as 0. Additionally, the death date variable was coded monthly from 2009 to 2015 to investigate the survival curve.

### Covariates

Demographic and chronic health conditions were used as covariates to calculate the adjusted hazard ratio (HR) in survival analysis. The covariates included age (middle-aged: <65 years, young-old and middle-old: 65–84 years, and old-old: ≥85 years), sex (male = 1; female = 0), smoking status (smoker = 1; non-smoker = 0), drinking (drinker = 1; non-drinker = 0), and presence of stroke and heart disease, hyperlipidemia, diabetes, and high cholesterol (present = 1; absent = 0). In terms of age, the subjects were divided into three groups: before older adults, young-old + old-old, and oldest-old.

### Statistical analysis

Demographic characteristics were analyzed using descriptive statistics. Survival analysis was performed to investigate the effect of physical activity, age, and BMI on survival rates in patients with dementia. Kaplan–Meier analysis was used to plot the median survival time and cumulative survival probability for each patient group [17]. The Kaplan–Meier method calculates the cumulative survival probability based on the probability of an event occurring after a certain point in time. Differences in survival rates between independent variable groups were examined using the log-rank test [17]. Although the Kaplan–Meier method allows for a visual comparison of differences in the likelihood of event occurrence between groups, it does not control for factors other than those specifically selected for analysis. Therefore, the Cox proportional hazards model was used for multivariate analysis to adjust for the study covariates and calculate HR [17]. The Cox proportional hazards model can analyze the impact of various attributes on the occurrence of a specific event. Descriptive statistics and survival analyses were performed using SAS software, version 7.1 (SAS Institute Inc, Cary, NC, USA), provided by the virtual server of the NHISS.

## Results

Table 1 shows the frequency distribution of the demographic characteristics of the study sample. The majority of the study sample were older adults with 65–84 years old (*n* = 4,852, 83.81%), female (*n* = 3,865, 66.76%), and almost half of the participants engaged in regular physical activity (*n* = 2,761, 47.69%). Among those who engaged in physical activity, the majority performed physical activity once or not at all during the past week. In terms of body weight, the participants were almost evenly distributed across normal weight (*n* = 2,151, 37.16%), overweight (*n* = 1,255, 21.68%), and obesity (*n* = 1,644, 28.40%). Most of the participants were non-smokers (*n* = 5,284, 91.28%) and non-drinkers (*n* = 5,326, 92.00%). Among the chronic diseases, hypertension was the most common (*n* = 3,372, 58.25%).

**Table 1:** Demographic characteristics (*n* = 5,789).

Variables	Frequency (%)
Age (years)	
Middle-aged, <65	345 (5.96)
Young-old and middle-old, 65–84	4,852 (83.81)
Old–old, ≥85	592 (10.23)
Sex	
Male	1,924 (33.24)
Female	3,865 (66.76)
Physical activity (Yes)	2,761 (47.69)
Physical activity frequency	
0	2,870 (49.58)
1	1,773 (30.63)
2	1,146 (19.80)
BMI	
Underweight	488 (8.43)
Normal weight	2,151 (37.16)
Overweight	1,255 (21.68)
Obesity	1,644 (28.40)
Severe obesity	251 (4.34)
Smoking	
Current smoker	505 (8.72)
Non-smoker	5,284 (91.28)
Drinking	
Current drinker	463 (8.00)
Non-drinker	5,326 (92.00)
Stroke	
Yes	719 (12.42)
No	5,070 (87.58)
Heart disease	
Yes	633 (10.93)
No	5,156 (89.07)
Hypertension	
Yes	3,372 (58.25)
No	2,417 (41.75)
Diabetes	
Yes	1,443 (24.93)
No	4,346 (75.07)
Hyperlipidemia	
Yes	283 (4.89)
No	5,506 (95.11)

BMI: Body mass index.

### Kaplan–Meier analysis and log-rank test

**Fig 2:**
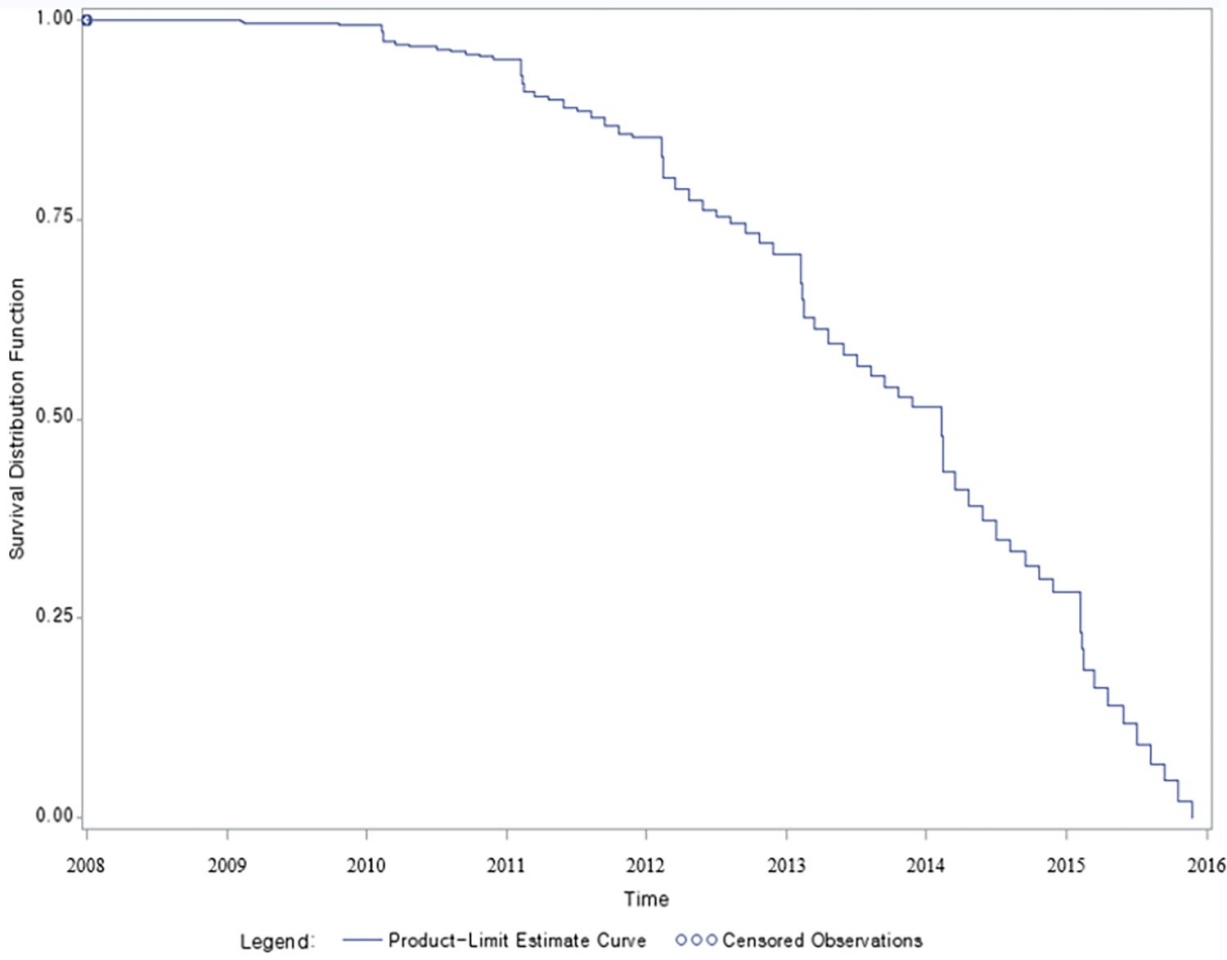
Kaplan–Meier survival curve.

This study investigated the time of death of patients with dementia based on a six-year dataset by plotting the Kaplan–Meier curve (Fig 2). The *x*-axis represents the death date for patients with dementia from 2009 to 2015, and the cumulative survival probability is plotted on the *y*-axis. A total of 5,789 patients were tracked, and 3,217 of them died between 2009 and 2015.

**Fig 3:**
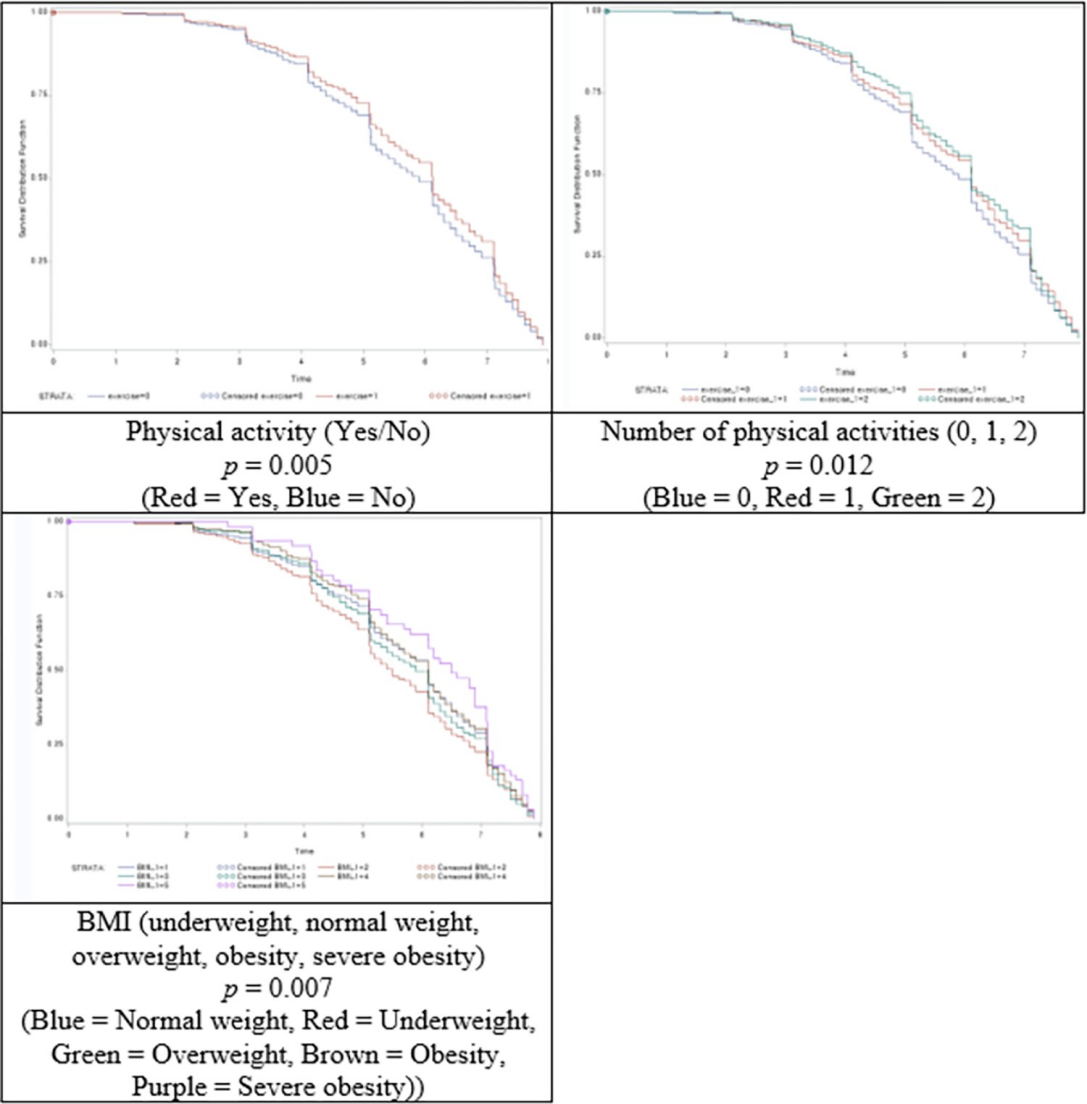
Log-rank survival curves.

Furthermore, this study estimated the time of death based on physical activity status, physical activity frequency, and BMI (Fig 3). The log-rank test revealed significant differences in physical activity status (*p* = 0.005), number of physical activities (*p* = 0.012), and BMI (*p* = 0.007).

### Cox proportional hazard model

Table 2 shows the results of the Cox regression analysis, with physical activity and BMI being the independent variables. Participants who engaged in physical activity had a significantly lower mortality risk (HR = 0.91, *p* = 0.02, 95% confidence interval [CI] = 0.84–0.99) than those who did not. Regarding body weight, participants with normal weight had a significantly lower mortality risk (HR = 0.86, 95% CI = 0.76–0.97, *p* = 0.01) than those who were underweight. Additionally, participants with obesity (HR = 0.85, 95% CI = 0.74–0.98, *p* = 0.03) and severe obesity (HR = 0.72, 95% CI = 0.55–0.95, *p* = 0.02) had a significantly lower mortality risk than those who were underweight. None of the other variables included in the analysis showed a significant association with mortality.

**Table 2:** Results of the Cox regression analysis of physical activity and BMI.

Variables	Estimate	HR	95% CI	*p*
Physical activity				
No	Ref.			
Yes	-0.09	0.91	0.84–0.99	0.02*
BMI				
Underweight	Ref.			
Normal weight	-0.15	0.86	0.96–0.97	0.01*
Overweight	-0.06	0.94	0.82–1.09	0.41
Obesity	-0.16	0.85	0.74–0.98	0.03*
Severe obesity	-0.33	0.72	0.55–0.95	0.02*
Age (years)				
<65	Ref.			
65–84	0.19	1.21	0.99–1.49	0.06
≥85	0.20	1.22	0.98–1.53	0.07
Sex				
Male	Ref.			
Female	-0.05	0.95	0.87–1.04	0.24
Smoking				
Current smoker	Ref.			
Non-smoker	0.09	1.10	0.96–1.24	0.17
Drinking				
Current drinker	Ref.			
Non-drinker	-0.04	0.96	0.83–1.11	0.57
Stroke				
Yes	Ref.			
No	-0.08	0.92	0.81–1.04	0.17
Heart disease				
Yes	Ref.			
No	-0.03	0.97	0.85–1.10	0.64
Hypertension				
Yes	Ref.			
No	0.03	1.03	0.95–1.12	0.43
Diabetes				
Yes	Ref.			
No	0.01	1.01	0.92–1.10	0.94
Hyperlipidemia				
Yes	Ref.			
No	0.13	1.14	0.91–1.42	0.24

CI: Confidence interval; HR: Hazard ratio; Ref.: Reference group.

*p < 0.05

**p < 0.001

***p < 0.0001.

Table 3 shows the subgroup analysis of the number of physical activities and mortality risk. Participants who engaged in 1 physical activity per week had a significantly lower mortality risk than those who did not (HR = 0.90, 95% CI = 0.82–0.99, *p* = 0.03). However, no significant difference was observed in mortality risk in participants who engaged in 2 physical activities per week (HR = 0.91, 95% CI = 0.81–1.01, *p* = 0.08).

**Table 3:** Results of Cox regression analysis of the number of physical activities.

Variables	Estimate	HR	95% CI	*p*
Physical activity frequency				
0	Ref.			
1	-0.10	0.90	0.82–0.99	0.03*
2	-0.10	0.91	0.81–1.01	0.08
BMI				
Underweight	Ref.			
Normal weight	-0.15	0.86	0.76–0.97	0.01*
Overweight	-0.06	0.94	0.82–1.09	0.41
Obesity	-0.16	0.86	0.74–0.99	0.03*
Severe obesity	-0.33	0.72	0.55–0.95	0.02*
Age (years)				
<65	Ref.			
65–84	0.19	1.21	0.99–1.48	0.06
≥85	0.20	1.22	0.98–1.52	0.08
Sex				
Male	Ref.			
Female	-0.05	0.95	0.87–1.04	0.25
Smoking				
Current smoker	Ref.			
Non-smoker	0.09	1.09	0.96–1.24	0.17
Drinking				
Current drinker	Ref.			
Non-drinker	-0.04	0.96	0.83–1.10	0.53
Stroke				
Yes	Ref.			
No	-0.08	0.92	0.82–1.04	0.18
Heart disease				
Yes	Ref.			
No	-0.03	0.97	0.85–1.10	0.64
Hypertension				
Yes	Ref.			
No	0.03	1.03	0.95–1.12	0.43
Diabetes				
Yes	Ref.			
No	0.01	1.00	0.92–1.10	0.94
Hyperlipidemia				
Yes	Ref.			
No	0.13	1.14	0.92–1.42	0.24

CI: Confidence interval; HR: Hazard ratio; Ref.: Reference group.

*p < 0.05

**p < 0.001

***p < 0.0001.

## Discussion

This study investigated the association of physical activity and BMI on the survival rates of patients with dementia. The Cox regression analysis was performed and revealed that participants who engaged in physical activity had a significantly lower mortality risk than those who did not. Participants with normal weight had a significantly lower mortality risk than those who were underweight, and participants with obesity or severe obesity had a significantly lower mortality risk than those who were underweight. No significant association was observed between other variables. These findings suggest that physical activity and body weight are important factors in determining the survival rates of patients with dementia.

The Cox regression analysis revealed that participants who engaged in physical activity had a significantly lower mortality risk than those who did not. Previous studies identified physical activity as a potential factor that may affect the dementia progression of patients with dementia [3,4]. Additionally, physical activity may improve cardiovascular function, reduce inflammation, and promote neuroplasticity, improving outcomes in patients with dementia [18]. These findings are consistent with the current recommendations that physical activity is essential for maintaining overall health and reducing the risk of chronic diseases such as dementia [19]. Previous studies and current recommendations support these findings.

However, in our study, no significant difference was observed in the risk of death among participants who engaged in physical activity twice a week. According to a study by Toots et al., [20], a high-intensity exercise program showed a significant decrease in ADL function and improved balance in dementia patients, while the exercise program showed cognitive decline. Additionally, de Souto Barreto et al. [21] reported that the intervention group which received exercise showed a decrease in cognitive function compared to the group that received music intervention and arts and crafts. They suggested that advanced age and excessive training might have contributed to this cognitive decline. Based on the evidence from these previous studies, it is believed that there was no significant difference in the risk of death among participants who engaged in physical activity twice a week as reported in our study.

This study found that BMI was associated with the survival rate of patients with dementia. Participants classified by BMI as normal weight, obese, or severely obese had a lower risk of death than underweight participants. These findings are consistent with previous research, which showed that being underweight is associated with an increased mortality risk in patients with dementia [22,23]. Weight loss may be a common symptom of dementia, which can further exacerbate the negative impact of low BMI on survival rates in patients with dementia [22]. Another study reported that being overweight or obese in middle age was associated with an increased risk of dementia but that being overweight or obese in later life did not increase the risk of dementia [10,12]. However, it may be related to the impact of BMI on overall health and immune function. This study highlights the importance of body weight in determining survival rates in patients with dementia, suggesting that maintaining a healthy weight may result in lower mortality in patients with dementia [8,24]. In addition, research suggests that being underweight, especially among older adults, may serve as a warning sign for compromised immune function and overall health. Winter et al. [25] found that both normal weight and higher BMI were associated with better health outcomes compared to underweight status among older adults. Thus, it is inferred that there was no discernible difference in outcomes between the commonly known unhealthy high BMI and normal BMI categories. This suggests that in older age groups, normal BMI and higher BMI may not yield significantly different results, as both represent a healthier state compared to being underweight. As another evidence, the studies conducted by Sobów et al. [26] provide compelling evidence that, within the context of mild cognitive impairment (MCI), the implications of BMI on health outcomes take on a different dimension. Specifically, these studies highlight that lower BMI and weight loss in individuals with MCI are associated with an increased risk of progression to dementia, including Alzheimer’s disease. This suggests that while higher BMI may generally be associated with better health outcomes in the broader elderly population, the situation is more complex for those with MCI. The findings imply that for MCI patients, maintaining a stable weight or avoiding underweight status could be particularly crucial in mitigating the risk of developing dementia. Therefore, it becomes evident that in the management and care of MCI patients, a nuanced approach to BMI and weight management is required, one that considers the unique risks and needs of this population.

Additionally, the control variables used for analysis in our study included age, sex, smoking, drinking, stroke, heart disease, hypertension, diabetes, and hyperlipidemia. Among them, only age showed a significant difference. In this study, based on previous research, age was classified into pre-older adult age, young-old + old-old, and oldest-old [27]. The risk of developing dementia tends to increase with age. According to Raz [28], the prefrontal cortex was reported to be the area with the greatest age-related vulnerability. Additionally, a study by Jorm, Jolley [29] reported that the incidence of dementia increases exponentially with age between 65 and 90 years and doubles approximately every 5 years. Similar to these previous studies, in this study, the risk of death in dementia patients increased as the age increased from 65 to 84 years old and over 85 years old compared to the under-65 age group.

This study has several limitations. First, the physical activity variable captured only a limited time range (e.g., during the past week). In addition, physical activity should encompass not just a wide time range but also include specific details regarding the duration of the activites being maintained. Second, because the data used in this study was from 2009 to 2015, generalization to 2023 may be difficult. Accordingly, future studies should utilize recent data and include variables including intensity and duration as well as time range of physical activity. Third, in this study, only BMI and physical activity were employed as crucial variables for analyzing the risk of mortality in patients with dementia. However, relying solely on these two variables is insufficient to meet the study’s validity requirements. This inadequacy stems from the omission of numerous other determinants associated with the risk of mortality in patients with dementia, including social and environmental determinants. The study, structured around variables of interest based on previous research, resulted in the exclusion of several decisive factors. Therefore, for future research endeavors, it is imperative to incorporate the latest data and explore variables related to the intensity, duration, and temporal aspects of physical activity. Additionally, there is a need to include social and environmental determinants associated with the risk of mortality in dementia patients. This approach will address the limitations of the current study and contribute to a more comprehensive understanding of the subject.

## Conclusion

This study reported that physical activity can reduce the risk of death in patients with dementia. However, there appeared to be no correlation with excessive exercise in patients with dementia. These results suggest that when implementing exercise programs for patients with dementia, it is advisable to tailor the exercise regimen based on the individual patient’s condition. Additionally, in this study, it was confirmed that among patients with dementia, normal weight compared to underweight, and overweight and obesity had a lower risk of death compared to underweight. Based on these results, it suggest that interventions are needed to adjust the diet of patients with dementia to maintain and increase sufficient nutrition and body weight. The study findings would be useful when establishing treatment programs for dementia patients.
